# Intestinal health of broilers challenged with *Eimeria* spp. using functional oil blends in two physical forms with or without anticoccidials

**DOI:** 10.1038/s41598-023-41743-9

**Published:** 2023-09-05

**Authors:** Priscila de Oliveira Moraes, Paula Gabriela da Silva Pires, Vilmar Benetti Filho, André Luís Ferreira Lima, Liris Kindlein, Diogo Taschetto, André Favero, Glauber Wagner

**Affiliations:** 1 Department of Animal Science and Rural Development, Universidade Federal de Santa Catarina, Florianópolis, Santa Catarina Brazil; 2 Advanced Poultry Gut Science, Florianópolis, Santa Catarina Brazil; 3 Laboratory of Bioinformatics, Center of Biological Sciences, Universidade Federal de Santa Catarina, Florianópolis, Santa Catarina Brazil; 4 Department of Preventive Veterinary Medicine, Universidade Federal do Rio Grande do Sul, Porto Alegre, Rio Grande do Sul 91540-000 Brazil; 5 Santa Livia Farm, Farroupilha, Rio Grande do Sul Brazil

**Keywords:** Biotechnology, Microbiology

## Abstract

This study aimed to assess the impact of a commercial blend of functional oils, specifically cashew nutshell liquid and castor oil (FO), in two physical forms (solid: P; liquid: S), in comparison to a combination of virginiamycin and anticoccidials on the gut health of broilers challenged with coccidiosis. A total of 1760 1-day-old male chicks were randomly distributed in a study design with eight treatments. The treatments included: a control group (without additive), OFS_0.75_kg/t (FO spray), OFP_1.0_kg/t (FO powder), OFP_1.5_kg/t (FO liquid spray), Sal (anticoccidials), Sal_Vir (virginiamycin and anticoccidials), Sal_OFS_0.5_ kg/t (anticoccidials plus FO spray), and Sal_OFP_1.0_kg/t (anticoccidials plus FO powder). All birds were challenged with *Eimeria* spp.* a*t 14 days. The physical form of FO did not affect performance and intestinal health parameters. At 42 days, broilers from the control and OFS_0.75 treatments were the lightest, while those from the Sal_Vir and Sal_OFP_1.0 treatments were the heaviest (P < 0.05). FO reduced the presence of *Clostridium perfringens*. The individual phytogenic additives did not prevent weight loss in birds challenged with *Eimeria*, but they mitigated the effects of the infection by modulating the intestinal microbiota. A synergistic effect was observed between the FO and anticoccidials, yielding satisfactory results in substituting virginiamycin.

## Introduction

*Eimeria,* the most economically impactful parasite in poultry, incurs a global cost of approximately USD 9.33 billion annually. This cost is attributed to prophylaxis, treatment, and production losses in broilers^1^. The severity of coccidiosis’s impact on intestinal integrity may be heightened in chickens that lack growth-promoting antibiotics. This is because the damage induced by coccidiosis provides substrates for specific bacteria to multiply, thereby disrupting the gut microbiome’s equilibrium. This disruption could potentially intensify malabsorption and enteritis, reduce growth rates, and increase feed conversion^2^.

The poultry industry has predominantly depended on ionophores for coccidiosis control. Ionophores, first licensed in 1971, have been extensively utilized for over 50 years, not only for combating coccidiosis but also as growth promoters. This is due to their antimicrobial properties, primarily against gram-positive bacteria. However, the intensive use of ionophores has led to the development of resistance among coccidia to this drug class^3^. Despite ionophores being exclusively used in animals and typically classified as feed additives, there is increasing public pressure to curtail or prohibit their use due to the growing bacterial resistance to antibiotics. The FDA categorizes ionophores as antibiotics, hence their prohibition in chickens labeled “Raised Without Antibiotics”^4^.

The decrease in the use of antibiotics as growth promoters has spurred the development of alternative methods to concurrently mitigate the effects of coccidiosis and enhance growth. A well-balanced intestinal microbiota is vital for optimal animal performance, particularly in the face of sanitary challenges. Research has shown that supplementing broilers, challenged with coccidiosis, with a commercial blend of functional oils, cashew nutshell liquid, and castor oil, results in a reduction of pathogenic bacteria such as *Clostridium perfringens*^5,6^. This reduction subsequently leads to improved performance.

This study aimed to assess the impact of supplementing a blend of functional oils, presented in two physical forms (powder or liquid spray), with or without ionophores*,* on the performance and intestinal health parameters of broilers challenged with *Eimeria* spp.

## Material and methods

The study adhered to protocol no. SL35_21 and received approval from the Ethics Committee on the Use of Animals of Granja Santa Lívia Produções e Pesquisa Agropecuária Ltda, under approval no. 007 (CIAEP 01.0627.2020). The research was conducted in strict compliance with the NIH Guide for the Care and Use of Laboratory Animals and was reported in line with the ARRIVE guidelines (https://www.nc3rs.org.uk/arrive-guidelines).

### Animals, facilities, and diets

A total of 1760 day-old male broiler chicks (Cobb 500) were distributed across 80 experimental floor pens (1.65 × 1.65 m, equipped with three nipple drinkers and one tubular feeder), each containing litter from three consecutive previous flocks. The oocyte count was not performed before 14 days of age, when the challenge was performed. Both food and water were provided ad libitum. The chicks were vaccinated against Marek's disease at the hatchery. The temperature and lighting regimens adhered to the recommendations of the broiler genetics supplier. The nutritional program was divided into three diets (Supplementary Table 1): starter (1 to 21 D), grower (21 to 35 D), and finisher (36 to 42 D), all based on the nutritional requirements suggested by the Brazilian Poultry and Swine Tables^7^. The treatments were assigned using a completely randomized design, comprising eight treatments with ten replicates, each containing 22 birds. The specifics of the treatments are outlined in Table 1. The eight treatments included: control (no additive); OFS_0.75 (blend of functional oils in liquid spray, 0.75 kg/t); OFP_1.0 (blend of functional oils in powder, 1.0 kg/t); OFP_1.5 (blend of functional oils in liquid spray, 1.5 kg/t); Sal_ (anticoccidials-nicarbazin + narasin 50:50 from 1 to 21 days, Salinomycin 66 ppm from 28 to 35 days and Salinomycin 60 ppm from 35 to 42 days); Sal_Vir (virginiamycin 16 ppm plus anticoccidials); Sal_OFS_0.5 (anticoccidials plus blend of functional oils in liquid spray, 0.5 kg/t); Sal_OFP_1.0 (anticoccidials plus blend of functional oils in powder, 1.0 kg/t) (Table 1).

**Table 1:** Description of treatments.

Abbreviation	Description	Powder	Spray
Control	Control^a^	–	–
OFS_0.75	Blend of functional oil^b^	–	0.75 kg/t
OFP_1.0	Blend of functional oil	1 kg/t	–
OFP_1.5	Blend of functional oil	1.5 kg/t	–
Sal	Anticoccidials^c^	–	–
Sal_Vir	Anticoccidials + virginiamycin 16 ppm	–	–
Sal_OFS_0.5	Anticoccidials + blend of functional oil	–	0.5 kg/t
Sal_OFP_1.0	Anticoccidials + blend of functional oil	1.0 kg/t	–

^a^Without AGP or another additive.

^b^Functional oil blend (Oligo Basics Agroind. Ltd., Cascavel, PR, Brazil; powder presentation: cardol: 5%; cardanol: 20%; ricinoleic acid: 9%; liquid spray presentation: cardol: 10%; cardanol: 40%; ricinoleic acid: 18%.

^c^Nicarbazin + narasin 50:50 from 1 to 21 days, salinomycin 66 ppm from 28 to 35 days and salinomycin 60 ppm from 35 to 42 days.

The basal diet was supplemented with various additives, as detailed in Supplementary Table 1. These included a functional oil blend in powder form (Oligo Basics Agroind. Ltd., Cascavel, PR, Brazil) with cardol at 5%, cardanol at 20%, and ricinoleic acid at 9%. The same blend in liquid spray form contained cardol at 10%, cardanol at 40%, and ricinoleic acid at 18%. That is, the mixture in spray form has twice as many active ingredients as the powder. Thus, although the dose of the powdered product is twice that of the spray, the concentration of the active ingredient will be the same.

Other additives were salinomycin at 12%, virginiamycin (both from Phibro Animal Health, Teaneck, NJ, US), and a combination of nicarbazin and narasin (Elanco, Indiana, US).

The spray product demonstrated a concentration of functional oils that was double that of the powder product. Therefore, the spray dose of 0.75 kg/t is equivalent to the 1.5 kg/t dose in the powder group. Similarly, the two groups that received a 1 kg/t dust dose are comparable to the 0.5 kg/t spray group.

### Coccidiosis challenge protocol

At the age of 14 days, birds were orally infected with 1 ml of a water suspension containing sporulated oocysts of *E. tenella* (5 × 10^3^), *E. acervulina* (2.5 × 10^4^), and *E. maxima (*2.0 × 10^4^)*. These oocysts* were procured from the CAPEV Laboratory (São Paulo, Brazil).

### Measurements

#### Production performance

Records of body weight and feed intake were taken at intervals of 7, 14, 21, 28, 35, and 42 days. The feed conversion was adjusted to compensate for the weight of deceased birds. Mortality was documented daily, noting the age and weight of the broilers, along with the most probable cause of death. The performance responses were categorized based on the following periods: pre-challenged (1–14 days of age), seven days post-challenged (14–21 days of age), 14 days post-challenged (21–28 days of age), the final period (28–42 days of age), and the total period (1–42 days of age).

#### Eimeria oocyst counts

Eimeria oocyst counts were determined at 14, 21, and 28 days of age. Excreta samples were collected from birds at five distinct entry points, thereby forming a pooled sample per pen. The oocyst count was ascertained using a McMaster chamber, employing the subsequent formula: total oocysts/g = (oocysts counted × dilution factor × [sample volume/counting chamber volume]/2).

#### Intestinal health index—‘I See Inside’

The ‘I See Inside’ (ISI) methodology (INPI BR1020150036019) is an index for assessing intestinal health. It employs the formula: ISI = Σ (EL × FI), where Σ denotes the sum, EL signifies the lesion score (ranging from 0 to 3, with 3 being the most severe) based on observed histological changes, and FI represents the pre-established Impact Factor (ranging from 1 to 3, with 3 being the most severe) determined by the effect on organ function. The ISI methodology evaluates several parameters for the intestine, including lamina propria thickness, epithelial thickness, enterocyte proliferation, infiltration of inflammatory cells in the epithelium and lamina propria, increase in goblet cells, congestion, and the presence of Eimeria oocysts^8^. At 21 and 28 days, one bird from each pen was euthanized via cervical dislocation. Jejunum samples were then collected, fixed in a buffered 10% formalin solution, and prepared in paraffin blocks. Hematoxylin and eosin (HE) staining was performed using alcian blue. Intestinal samples were assessed by analyzing five villi per bird with a Leica DM1000 LED optical microscope. For each bird, a total of 20 villi and 20 adjacent crypt depths were analyzed.

#### Microbiome

Rectal contents were collected from three birds per pen at 14 and 21 days of age through abdominal pressure. These samples were homogenized, pooled, and immediately stored at -20 °C, with ten replicates for each treatment. DNA extraction was performed using the QIAamp DNA Stool Mini kit (QIAGEN, Hilden, Germany), adhering to the manufacturer’s guidelines. The 16S rRNA V3/V4 region was amplified using the 341F (5′-CCTACGGGRSGCAGCAG-3') and 806R (5′-GGACTACHVGGGTWTCTAAT-3′) primers, with Illumina adapters for sequencing. The amplification process involved 35 cycles at an annealing temperature of 50 °C, and was tripled for each sample. Sequencing was conducted using the Illumina MiSeq V2-500 kit in a 500-bp paired-end run. The raw sequences were subsequently uploaded to the Sequence Read Archive (SRA) under the bioproject number PRJNA882262.

### Bioinformatics analysis

The sequences underwent processing via a proprietary pipeline (Neoprospecta Microbiome Technologies, Brazil). In brief, Illumina adapters and sequences of low quality (Phred Quality < 20) were eliminated, and the remaining sequences were clustered with 100% identity. These clusters were excluded from further analysis of putative chimeric sequences. Additionally, clusters containing fewer than two sequences were also omitted. Amplicon sequence variants (ASVs) were classified using blastn v2;6;0+ in conjunction with a precise 16S rRNA database derived from the National Center for Biotechnology Information (NCBI/Genbank-NeoRefdb; Neoprospecta Microbiome Technologies^®^). The taxonomic results were determined at the species level.

Relative abundance, as well as alpha (Chao-1, Shannon, and Simpson) and beta diversity indices were calculated using the R program (v. 3.6.1) (https://www.R-project.org/) with the reshape2 (v. 1.4.4)^9^ and phyloseq (v. 1.38.0)^10^ packages. The estimation of beta diversity was conducted post-normalization of the sequence number, achieved by randomly selecting an equal number of sequences from each sample. After normalization, Uniform Manifold Approximation and Projection (UMAP) was performed using the Bray–Curtis dissimilarity index in the vegan package (v. 2.5.7)^11^.

### Statistical analysis

Treatments were distributed in a completely randomized block design, comprising eight treatments and 10 replicates, each with 22 birds per experimental unit. Data were statistically analyzed using SAS 9.0, with the normality of variance tested before proceeding with further analysis. Significance was considered when the probability was lower than 5%.

Excretion of oocysts did not follow a normal distribution were evaluated using the Kruskal–Wallis non-parametric test. The Wilcoxon test was employed for pairwise treatment comparisons, with corrections made for the false discovery rate. The beta diversity distances were compared using the Adonis test.

Sequences and abundance taxonomy profiles were used to predict metabolic pathways using Phylogenetic Investigation of Communities by Reconstruction of Unobserved States (PICRUSt2 v2.4.2) using default settings. The predicted pathways were then summarized in level 2 and 3 (L2 and L3) of Kyoto Encyclopedia of Genes and Genomes (KEGG)^12–14^ pathways. Subsequently, these pathways were analyzed using STAMP 2.1.3 to compare treatments at the L2 and L3 levels. Multiple groups were compared using the Kruskal–Wallis H-test followed by a post-hoc Turkey–Kramer correction using the False Discovery Rate (FDR, Benjamini–Hochberg). Pairwise tests were performed using a two-sided Welch t-test with an FDR correction (Benjamini–Hochberg) for multiple comparisons. A corrected p-value limit of 0.05 was established as the significance value.

## Results

### Performance

As mentioned in material and methods the performance responses were categorized based on the following periods: pre-challenged (1–14 days of age), seven days post-challenged (14–21 days of age), 14 days post-challenged (21–28 days of age), the final period (28–42 days of age), and the total period (1–42 days of age). Table 2 presents data on body weight gain (BWG), daily feed intake (FI), and feed conversion rate (FCR). No significant differences were detected in any of the analyses when the physical form factor of the product was evaluated independently (data not shown). Similarly, no statistical difference was found in mortality rates. The average mortality rate throughout the experiment remained below 5% (data not shown).

**Table 2:** Live weight, weight gain per period (WG), feed intake (FI), and feed conversion ratio (FCR) of broilers in the period of 1 to 42 days of age.

Variable	Control^1^	OFS_0.75	OFP_1.0	OFP_1.5	Sal	Sal_VIR	Sal_OFS_0.5	Sal_OFP_1.0	SEM	Prob^2^
1–14 days (pre-infection)										
BWG	32.50^ab^	32.38^ab^	32.60^ab^	33.06^a^	32.51^ab^	33.47^a^	32.07^b^	32.47^ab^	0.11	***
FI	36.70	36.96	36.73	37.27	36.80	37.07	36.30	36.62	0.10	ns
FCR	1.13^ab^	1.14^a^	1.13^ab^	1.13^ab^	1.13^ab^	1.11^b^	1.13^ab^	1.13^ab^	0.002	*
14–21 days (7 days post-infection)										
BWG	52.91^b^	53.48^b^	53.0^b^	53.45^b^	63.09^a^	65.00^a^	62.91^a^	64.00^a^	0.75	*
FI	83.35^b^	83.86^b^	82.67^b^	80.34^b^	92.19^a^	92.90^a^	90.46^a^	91.54^a^	0.73	*
FCR	1.58^a^	1.57^a^	1.54^ab^	1.51^abc^	1.46^bc^	1.43^c^	1.44^c^	1.43^c^	0.009	*
21–28 days (14 days post-infection)										
BWG	85.51^c^	87.98^bc^	88.54^bc^	90.04^ab^	91.55^ab^	95.62^a^	92.40^ab^	93.00^ab^	0.63	*
FI	138.70	140.75	140.21	142.20	142.90	145.11	142.17	142.89	0.61	ns
FCR	1.62^a^	1.60^ab^	1.58^abc^	1.58^abc^	1.58^abc^	1.52^c^	1.54^bc^	1.54^bc^	0.006	*
28–42 days (final period)										
BWG	120.13	121.46	122.76	120.79	123.23	123.11	124.61	125.06	0.47	ns
FI	212.29	211.01	211.55	208.06	211.91	209.48	212.71	212.48	0.75	ns
FCR	1.77^a^	1.74^ab^	1.72^b^	1.72^b^	1.72^b^	1.70^b^	1.71^b^	1.70^b^	0.004	*
1–42 days (total period)										
BWG	73.95^d^	74.85^d^	75.53^bc^	75.20^c^	77.69^abc^	78.96^a^	78.11^ab^	78.68^a^	0.30	*
FI	120.01	120.09	119.91	118.87	122.39	121.85	121.78	122.11	0.36	ns
FCR	1.62^a^	1.60^ab^	1.59^bc^	1.58^bcd^	1.58^cde^	1.54^f^	1.59^def^	1.55^ef^	0.003	*

Means of 22 birds per pen, 10 replicates per treatment, 220 birds per treatment. Birds will be challenged orally at 14 days of placement with a coccidiosis inoculum.

*SEM* standard errors of the mean.

^1^NC: without AGP or another additive, PC: virginiamycin 16 ppm and salinomycin 66 ppm, PC2: salinomycin 66 ppm, NC_FOP_1.5: NC + functional oil powder, NC_FOS_0.75: NC + functional oil spray, PC2_FOP_1: PC2 + functional oil powder, PC2_FOS_0.5: PC2 + functional oil spray (PC2_FOS_0.5), NC_FOP_1: NC + functional oil powder.

^2^Probabilities, means with different letters differ statistically by Tukey: 0 ‘***’ 0.001 ‘**’ 0.01 ‘*’ 0.05.

In the pre-challenged period, the body weight gain (BWG) and live weight (LW) at 14 days of the Sal_Vir and OFP_1.5 groups outperformed the Sal_OFS_0.5 group (P < 0.05), with other treatments demonstrating intermediate values. However, by the 21st day, all treatments lacking anticoccidial supplementation underperformed compared to those with anticoccidial supplementation. Consequently, treatments supplemented solely with functional oils, irrespective of dosage or physical form, and without anticoccidials, did not show any significant difference from the control treatment. They performed worse than the treatments with anticoccidials, which, in contrast, showed no significant difference amongst themselves (P < 0.05).

**Figure 1:**
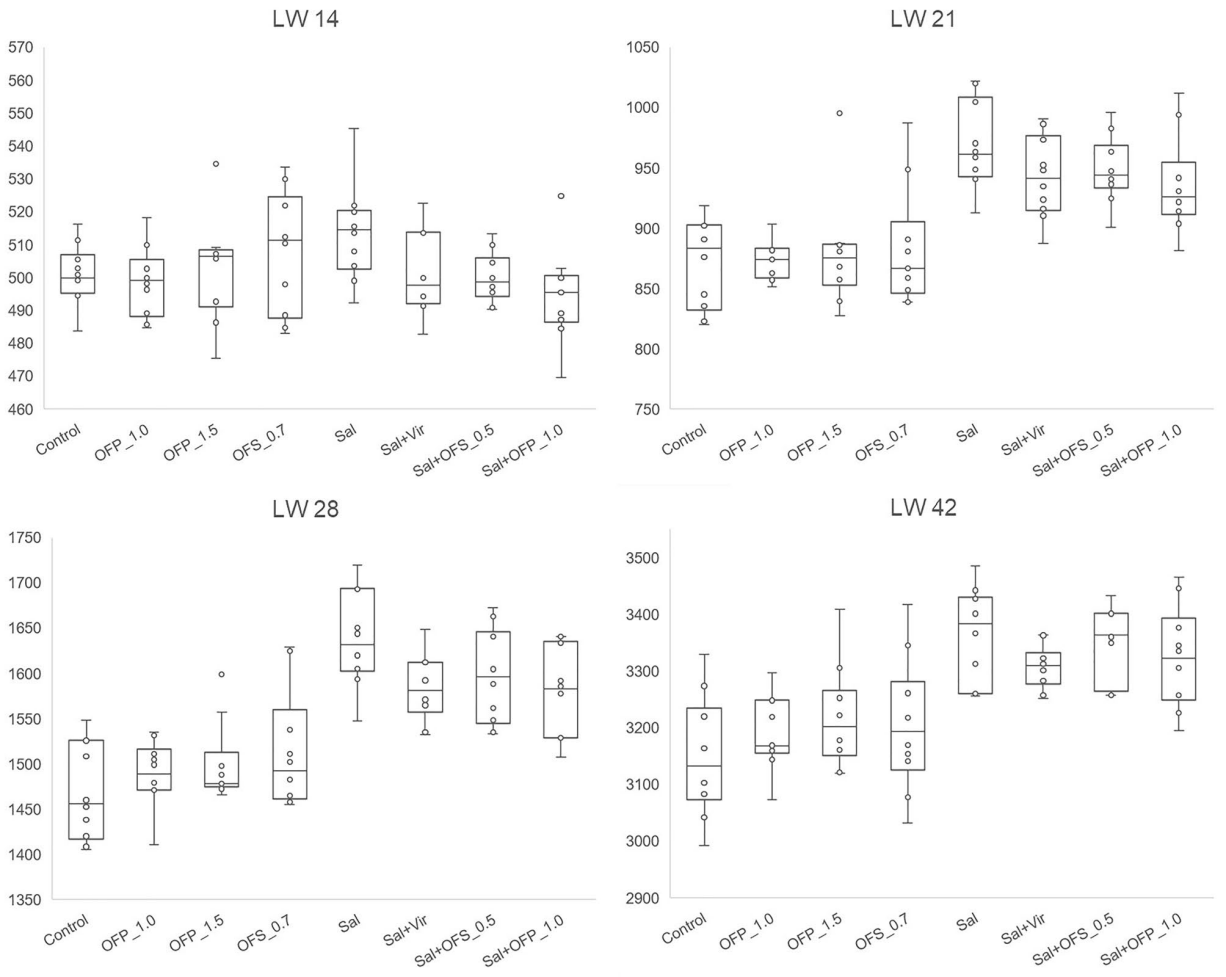
Boxplot of live weight of broilers in the pre-infection, 7 days after infection, 14 days after infection, and at 42 days of life.

Fourteen days post-challenge, no statistical difference in FI was observed across treatments. The BWG was significantly higher in the Sal_Vir treatment and lower in the control (P < 0.05). While the anticoccidial treatments did not differ from the OFP_1.5 treatment, the lower dosages of the functional oil blends (OFS_0.75 and OFP_1.0) were intermediate. They did not differ from the control treatment but were lighter than the Sal_Vir treatment (P < 0.05). In the final period (28–42d), neither FI nor BWG showed any differences. However, the FCR was worse in the control treatment compared to all other treatments, except for the OFS_0.75 treatment, which did not differ from any other treatment. Feed intake was not different among treatments at 42 D. However, the BWG at this age was the lowest for the control and OFS_0.75 treatments, being lower than the Sal_Vir and Sal_OFP_1.0 treatments (P < 0.05), which exhibited the highest BWG and did not differ from each other. The BWG for the OFP_1.5 treatment was higher than the control (P < 0.05), and lower than the Sal_Vir, Sal_OFS_0.5, and Sal_OFP_1.0 treatments (P < 0.05), not differing from the Sal treatment. The LW results at 42 days were similar to those of the BWG (Fig. 1).

### Oocysts analysis

At 14 days of age, the birds were intentionally infected with a pool of *Eimeria* spp. Upon examination of the bedding at 21 days, it was found to be contaminated, albeit at a lower concentration. This contamination likely occurred due to the reuse of the litter, as outlined in the materials and methods section. To clarify the timeline, the term “pre-challenged” refers to the period before the planned infection at 14 days of age, “peak of challenged” to the 21-day mark, and “post-challended” to the period following 28 days of age.

**Figure 2:**
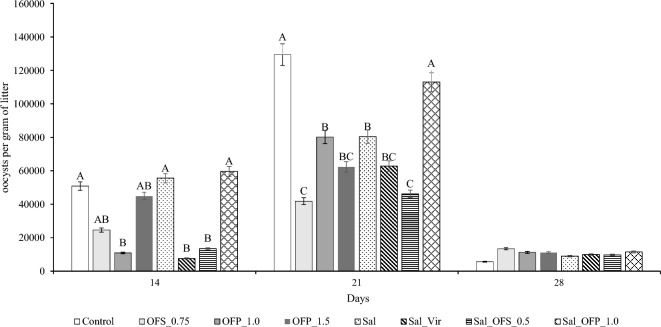
Excretion of oocysts in broilers challenged with coccidiosis in the pre-challenged period (14 D), 7 days after infection (21 D), and 14 days after infection (28 D). Different capital letters demonstrate differences within the period with the Kruskal–Wallis non-parametric test.

There was no significant difference observed in oocyst counts across all treatments at 28 d of age. However, at 14 and 21 days, statistical differences were noted among the treatments as per the Kruskal–Wallis test. The control, Sal, and Sal_OFP_1.0 treatments exhibited higher tendencies of oocyst counts at 14 days compared to the OFP_1.0, Sal_Vir, and Sal_OFS_0.5 treatments. The OFS_0.75 and OFP_1.5 treatments demonstrated intermediate tendencies in oocyst counts (Fig. 2). At 21 days, the Control and Sal_OFP_1.0 treatments showed higher tendencies of oocyst counts. The lowest oocyst counts were observed in the OFP_1.5 and Sal_OFS_0.5 treatments compared to the others. All treatments followed the same shedding patterns in oocyst counts, with the highest count at 21 days (seven days post-challenge) and the lowest at 28 days (fourteen days post-challenge).

### Intestinal health index—‘I See Inside’

The correlation between villus height and crypt depth at both 21 and 28 days was lower in Sal, Sa_Vir, and the control treatment compared to OFP_1.5 and OFS_0.75 (P < 0.05). The remaining treatments demonstrated intermediate results (Table 3).

**Table 3:** Intestinal histology and Intestinal Health Index of broilers at 21 and 28 days of age.

Treatments^1^	21 days				28 days			
	Villus height	Crypt depth	V:C	ISI	Villus height	Crypt depth	V:C	ISI
Control	869^abc^	188^a^	5.03^c^	15.39^a^	950^bc^	155^ab^	6.98^b^	9.84^ab^
OFS_0.75	899^ab^	176^ab^	5.79^a^	12.97^c^	985^b^	153^ab^	7.27^a^	9.17^abc^
OFP_1.0	854^bc^	170^b^	5.61^ab^	13.37^bc^	937^ cd^	166^a^	6.47^ab^	10.18^a^
OFP_1.5	904^a^	174^ab^	5.71^a^	11.91^c^	1043^a^	163^a^	7.20^a^	8.60^bc^
Sal	785^d^	175^ab^	4.94^c^	14.85^ac^	869^ef^	140^b^	7.05^b^	8.54^bc^
SAL_VIR	736^e^	168^b^	4.76^c^	13.21^ab^	846f.	152^ab^	6.27^b^	6.72^d^
Sal_OFS_0.5	825^ cd^	169^b^	5.33^b^	15.48^a^	902^de^	162^a^	6.40^ab^	8.90^abc^
Sal_OFP_1.0	801^d^	174^ab^	5.11^b^	15.70^a^	987^b^	162^a^	6.90^ab^	7.77^d^
SEM	116.7	50.38	1.4	0.10	106.87	30.80	1.6	0.05
P-value^2^	< 0.0001	< 0.0001	0.004	< 0.0001	< 0.0001	< 0.0001	0.009	< 0.0001

Means of 10 birds per treatment. Birds will be challenged orally at 14 days of placement with a coccidiosis inoculum.

*SEM* standard errors of the mean.

^1^NC: without AGP or another additive, 2 PC: virginiamycin 16 ppm and salinomycin 66 ppm, PC2: salinomycin 66 ppm, NC_FOP_1.5: NC + functional oil powder, NC_FOS_0.75: NC + functional oil spray, PC2_FOP_1: PC2 + functional oil powder, PC2_FOS_0.5: PC2 + functional oil spray (PC2_FOS_0.5), NC_FOP_1: NC + functional oil powder.

^2^Probabilities, means with different letters differ statistically by Tukey 0.05%.

At 21 days, the ISI index was observed to be higher in the control, Sal_OFS_0.5, and Sal_OFP_1.0 treatments compared to the OFS_0.75 and OFP_1.5 treatments (P < 0.05), with the remaining groups presenting intermediate values. Upon further examination of the ISI results, it was noted that the presence of oocysts was lower in treatments involving only functional oils (OFS_0.75, OFP_1.0, and OFP_1.5) compared to the Sal_OFP_1.0 treatment, which exhibited the highest rates (P < 0.05) (Supplementary Table 2).

At 28 days, the index for Sal_Vir and Sal_OFP_1.0 treatments was lower compared to other treatments. At this stage, the presence of oocysts did not differ. However, Sal_Vir exhibited the lowest values for lamina propria thickness, epithelial thickness, enterocyte proliferation, and inflammatory cell infiltration in the epithelium. These values were comparable to those of Sal_OFP_1.0 and Sal_OFS_0.5. For these variables, both anticoccidials and FO alone exhibited higher values, but these were not significantly different from the controls.

### Alfa e beta diversities

**Figure 3:**
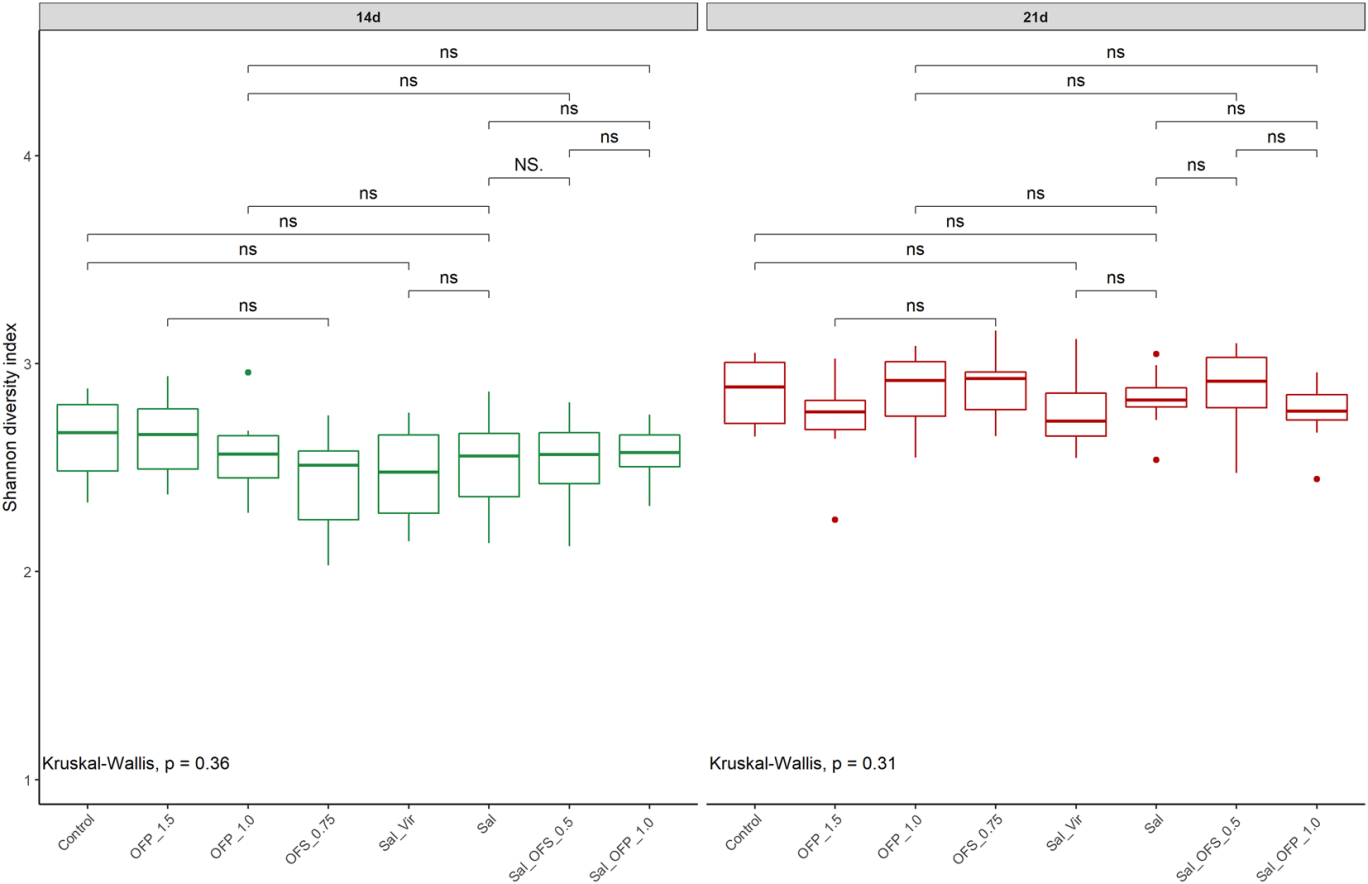
Comparison of alpha diversity between additives with Shannon index at 14 and 21 days. Control: without additive; OFS_0.75: blend of oil functional spray 0.75 kg/t; OFP_1.0: blend of oil functional powder 1.0 kg/t; OFP_1.5; blend of oil functional spray 1.5 kg/t; Sal: salinomycin 66 ppm; Sal_Vir: virginiamycin 16 ppm and Sal 66 ppm; Sal_OFS_0.5: Sal 66 ppm plus blend of oil functional spray 0.5 kg/t; Sal_OFP_1.0: Sal 66 ppm plus blend of oil functional powder 1.0 kg/t.

**Figure 4:**
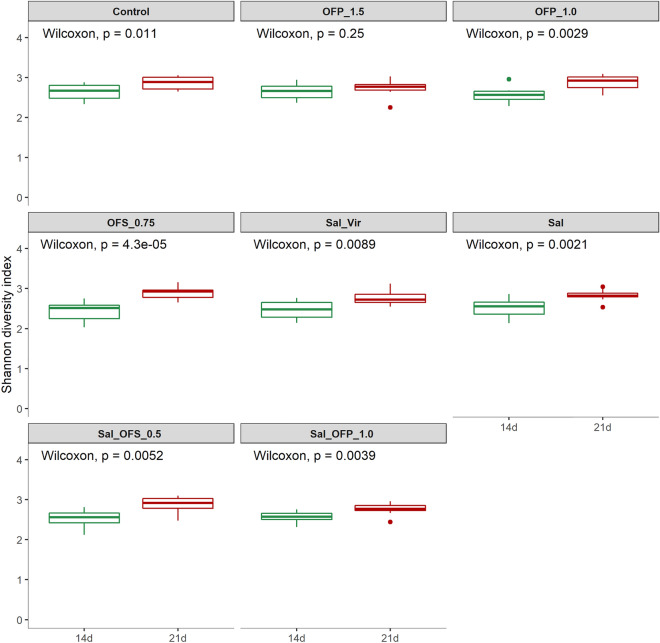
Comparison of alpha diversity for each additive between times with Shannon index. Control: without additive; OFS_0.75: blend of oil functional spray 0.75 kg/t; OFP_1.0: blend of oil functional powder 1.0 kg/t; OFP_1.5; blend of oil functional spray 1.5 kg/t; Sal: salinomycin 66 ppm; Sal_Vir: virginiamycin 16 ppm and Sal 66 ppm; Sal_OFS_0.5: Sal 66 ppm plus blend of oil functional spray 0.5 kg/t; Sal_OFP_1.0: Sal 66 ppm plus blend of oil functional powder 1.0 kg/t.

A total of 3,166,356 high-quality sequences were identified, averaging 19,789.72 ± 9535.18 per sample, with a plateau discovery at the species level, as depicted in the rarefaction curve (Supplementary Fig. 1). At 14 days, the Sal_OFS_0.5 treatment exhibited greater diversity compared to Sal, and the OFS_0.5 treatment showed higher diversity than Sal_OFP_1.0, but only in the CHAO 1 index (P < 0.05) (Supplementary Fig. 2a). No significant differences were observed in the Shannon and Simpson indices among treatments at 14 and 21 days (Fig. 3 and Supplementary Fig. 2). When comparing treatments over time, all treatments, except for OFP_1.5, showed increased diversity at 21 days in the Simpson and Shannon indices. For the CHAO 1 index, only treatments with Sal and OFS_0.75 increased diversity at 21 d (P < 0.05). (Fig. 4 and Supplementary Fig. 3a,b).

**Figure 5:**
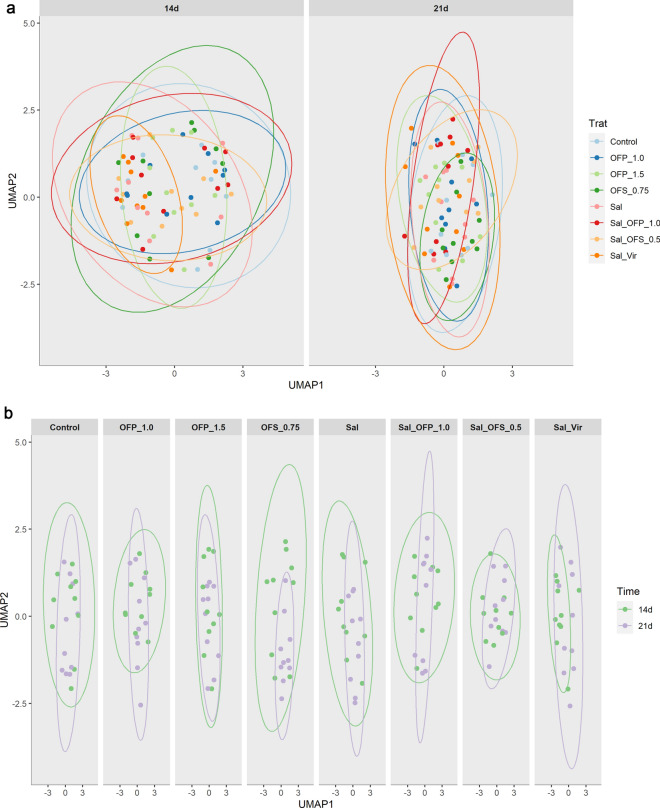
Uniform Manifold Approximation and Projection (UMAP) of beta diversity based on Bray–Curtis dissimilarity. (**A**) Comparison between all additives by time. (**B**) Comparison for each additive at different times.

We calculated Bray–Curtis dissimilarity values to quantify individual variations in taxonomic structure. To illustrate the disparities among the microbiome profiles, we executed UMAP on the Bray–Curtis dissimilarity matrix. However, this did not yield a distinct clustering pattern. All samples were dispersed with intersecting ellipses at 14 and 21 days (Fig. 5a). Upon comparing each treatment at varying ages, we discovered that the distribution of the paired beta diversity values remained consistent. This consistency was observed at both ages (Fig. 5b, Supplementary Table 3).

### Relative abundance and potential function of the gut microbiome

**Figure 6:**
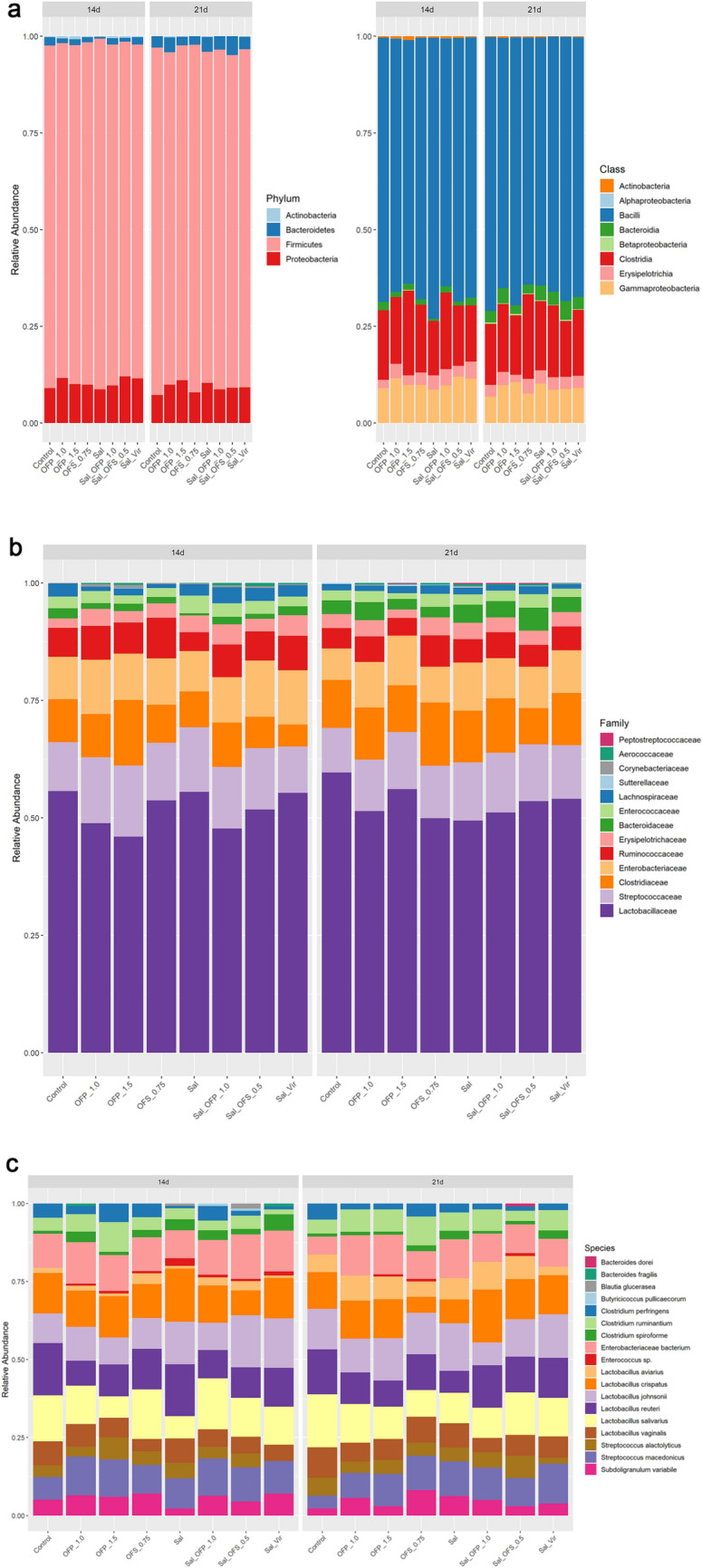
Relative abundance of broilers rectal samples at 14 and 21 days. Presented in total percentage (%) at Phylum and Class taxonomic level (**A**), Family level abundances greater than 1% (**B**) and Species level abundances greater than 5% (**C**).

We conducted a comprehensive analysis of the taxonomic composition, including the phylum, class (Fig. 6a), family (Fig. 6b), and species with abundances exceeding five percent (Fig. 6C) (Refer to Supplementary Table 4). The Firmicutes phylum was predominant in all treatments and at both ages, accounting for approximately 87% of the composition. This was followed by Proteobacteria (~ 9.64%) and Bacteroidetes (~ 2.42%). Families that had an abundance greater than 1% across all treatments included *Lactobacillaceae* (~ 52%), *Streptococcaceae* (~ 12%), *Clostridiaceae* (~ 9.77%), *Enterobacteriaceae* (9.43%), *Ruminococcaceae* (5.83%), *Lachnospiraceae* (1.84%), *Enterococcaceae* (2.42%), *Bacteroidaceae* (3.27%), and *Erysipelotrichaceae* (3.27%). Notably, there was a variation in the relative abundance of Lactobacillus species between collection times. Species such as *L. reuteri, L. salivarius, L. crispatus, L. johnsonii,* L. *helveticus*, and *L. aviarius* showed a decrease*.* Conversely, *C. perfringen*s increased in all treatments, with the exception of OFP_1.0, OFP_1.5, and Sal_OFP_1.0.

The relationship between the host and intestinal microorganisms and its impact on broiler performance was examined using PICRUSt2 to investigate the functional profiles of the microbiota.

**Figure 7:**
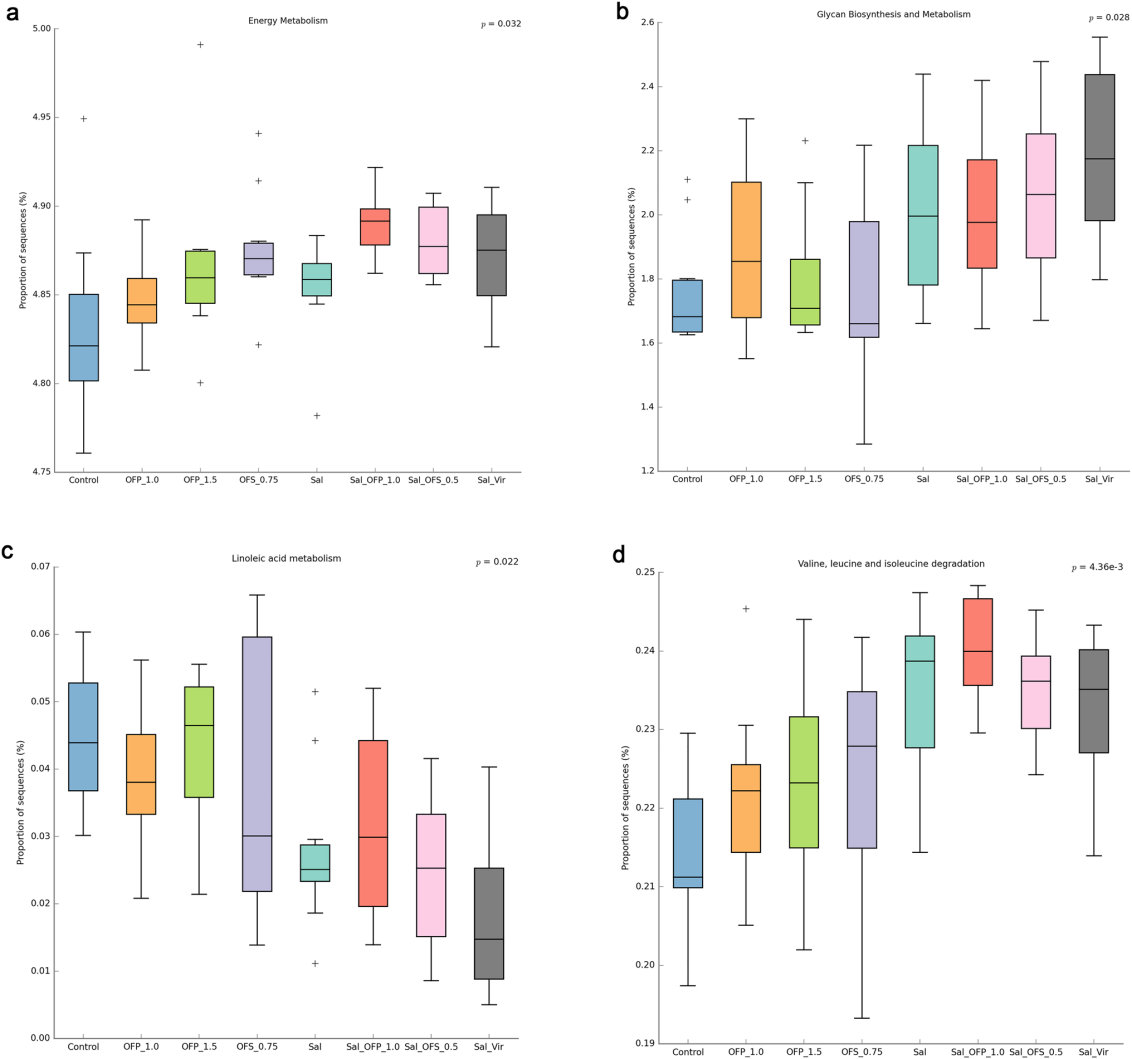
Proportion of sequences for each metabolic pathway at level 2 (L2) and 3 (L3) of KEGG, predicted by PICRUSt2, visualized with STAMP. Multiple comparisons were evaluated by Kruskal–Wallis H-test, corrected by false discovery rate. (**A**) KEGG L2 energy metabolism between additives at 21 days. (**B**) KEGG L2 glycan biosynthesis between additives at 21 days. (**C**) KEGG L3 linoleic acid metabolism between additives at 21 days. (**D**) KEGG L3 Valine Leucine and Isoleucine degradation between additives at 21 days.

Upon observing the biosynthesis and metabolism of glycans, as well as the degradation of the amino acids valine, leucine, and isoleucine, it was found that the scores for treatments supplemented with anticoccidials were higher. This contrasted with the metabolism of linoleic acid, which displayed lower values. In the case of L2 pathways, the Sal_OFP_1.0 scores were elevated for functional genes associated with energy metabolism (Fig. 7a–d).

**Figure 8:**
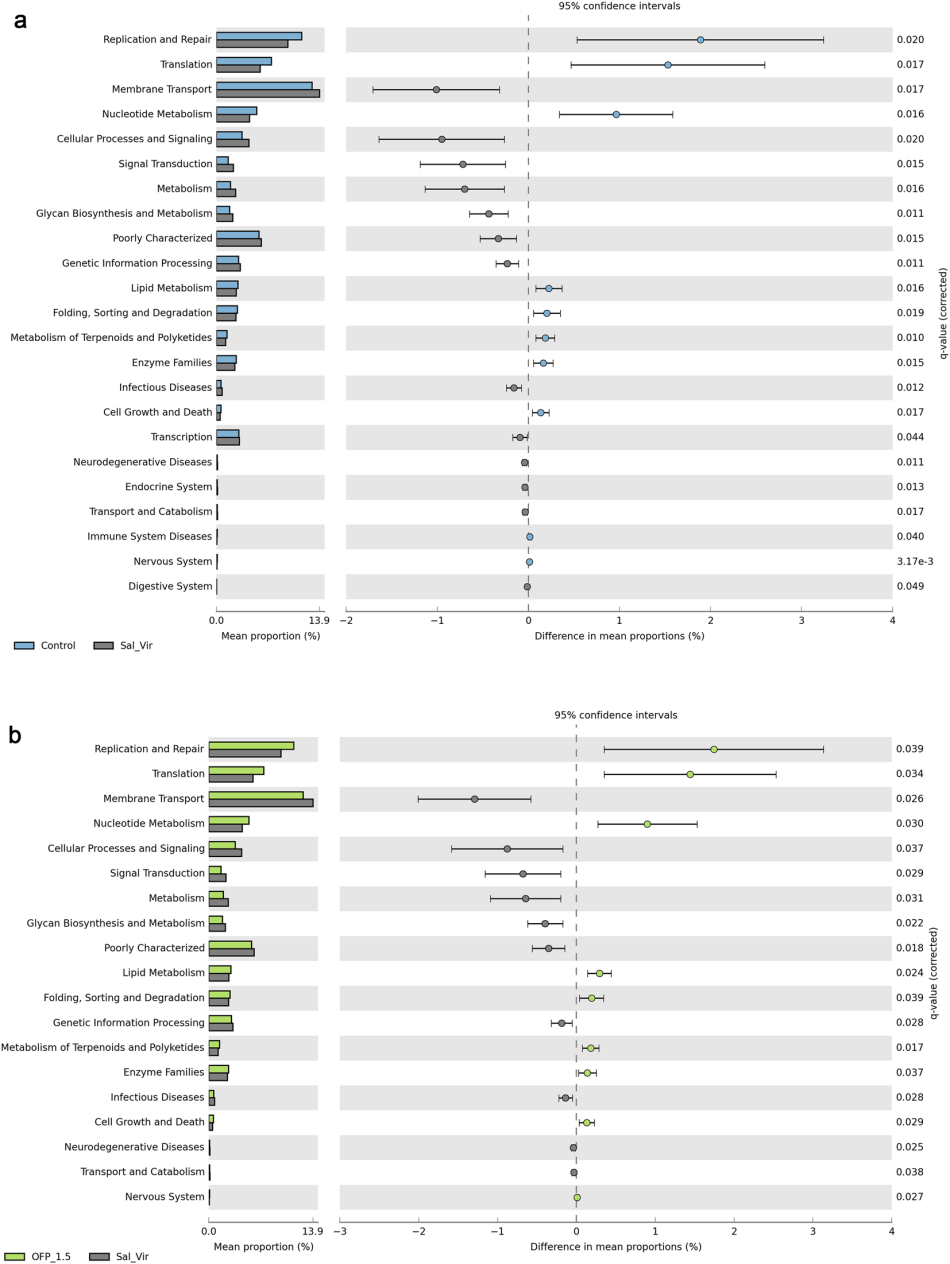
Comparision of mean proportion of sequences with extended error bar of predicted L2 kegg pathways at 21 days between (**A**) control and Sal_Vir; (**B**) OFP_1.5 and Sal_Vir. Pairwise comparisions were made using Welch t test, corrected by FDR.

A pairwise comparison of KEGG L2 pathways at 21d between the control treatment and anticoccidials revealed a higher percentage of replication and repair in the control treatment. Conversely, anticoccidials exhibited enhanced metabolism, biosynthesis, and metabolism of glycans (Fig. 8a). A similar comparison at 21d between Sal and OFP_1.5 indicated a higher score for Sal in terms of biosynthesis and metabolism of glycans, metabolism, and membrane transport. On the other hand, OFP_1.5 demonstrated a superior metabolism of lipids, terpenoids, and nucleotides (Fig. 8b).

## Discussion

The combination of anticoccidials and the antibiotic Sal_Vir improved broiler performance throughout the experiment, resulting in lower oocyst excretion. However, this combination also led to a decreased villous:crypt ratio and an increased ISI. The powdered functional oil blend, when used alone at a concentration of 1.5 kg/t, yielded intermediate performance results, surpassing the control group with a higher villous:crypt ratio and a lower ISI. Although the phytogenic additive did not prevent weight loss in Eimeria-challenged birds, it mitigated the effects of the infection. The results indicated that the combination of anticoccidials and the powdered functional oil blend at 1 kg/t (Sal_OFP 1.0) enhanced animal performance, mirroring the performance of Sal_Vir in terms of average daily weight gain from 1 to 42 days of life, with respective gains of 78.68 g and 78.96 g. It is noteworthy that the physical forms of the functional oil blends did not affect performance when used at the same concentration. Ionophores either used alone or in combination, are commonly utilized in global poultry coccidiosis prevention programs. While virginiamycin is not an anticoccidial drug, its efficacy as a performance-enhancing antibiotic is well-established. Broilers challenged with *E. maxima* performed better when supplemented with virginiamycin, although the exact mechanisms of action remain unclear ^15^. One potential contributing factor could be the reduced activity of the immune system, as indicated by the decreased expression of pro-inflammatory cytokines. In this study, we observed that Sal_Vir treatment at both 21 and 28 days resulted in a thinner epithelium and lamina propria, reduced infiltration of inflammatory cells in the epithelium and lamina propria, and decreased proliferation of goblet cells (Supplementary Table 3).

Contrarily, these factors did not diminish the presence of oocysts in the epithelium at 21 days; in fact, the highest rates were noted in treatments with anticoccidials, while treatments supplemented solely with Sal_Vir exhibited the lowest. The modulation of the immune system, which enhances broiler resilience and bolsters the immune response against the pathogen^5^, is of significant importance. When the mucosa is subjected to aggression, cell replacement occurs, consuming nutrients derived from the chicken’s energy reserves and ingested diet. It is projected that the upkeep of the intestinal epithelium and its supporting structures consumes 20% of the energy intake of the animal^16^, thereby negatively impacting animal performance.

Broilers that did not receive anticoccidial treatments required a more substantial immune response compared to those that were supplemented, due to the parasite-eliminating properties of the ionophore. The prophylactic application of ionophores has significantly propelled the growth of the poultry industry^17^. Research has documented the positive impact of anticoccidials, such as salinomycin, on the performance of broilers when challenged with *Eimeria* spp^18^. The rotation of ionophores is one of the most effective strategies to maintain anticoccidial efficacy and mitigate resistance^8^. Cross-resistance can also occur between ionophores of the same class in the field; thus, alternating different classes of ionophores or employing phytogenics can prevent the emergence of resistant coccidia. Studies involving phytogenics have demonstrated promising results in broiler performance when faced with coccidiosis^5,18,19^.

The impact of parasites on enterocytes modifies the intestinal environment and the diversity of the microbiota. Various studies have indicated that this modification can either increase ^19^ or decrease ^20^ diversity. In this study, the litter used for the experiment was reused, which likely resulted in low contamination by *Eimeria* spp. prior to the challenge. Although the litter was uniformly distributed in the pens, not counting the number of oocysts before placing the birds can be a limiting factor for discussing the results, since there is no information on the number of oocysts before 14 days of age. Consequently, the oocyst was detected before the scheduled challenge. It was noted that the treatment with OFP_1.5 preserved the diversity of the microbiota before the challenge (14 d) and at the peak of the infection (21 d), unlike the other treatments. This observation supports the results found by previous studies^21^, demonstrating the modulating effect of the microbiota in response to the challenge.

Following a coccidiosis infection, a decrease in the relative abundance of certain families, including *Ruminococcaceae*, *Lachnospiraceae, Erysipelotrichaceae*, and *Aerococcaceae*, was observed. Conversely, an increase was noted in *Lactobacillaceae, Clostridiaceae*, and Enterobacteriaceae, compared to the observations made at 14 and 21 days of age. The families *Ruminococcaceae, Lachnospiraceae*, and *Erysipelotrichaceae* are anaerobic bacteria involved in the fermentation of structural carbohydrates and the production of short-chain fatty acids. In contrast to other treatments, Sal_Vir led to an increase in the relative abundance of the *Ruminococcaceae* family. On the other hand, OFP_1.5 and FOS_0.75 resulted in an increase in the relative abundance of *Lachnospiraceae* and *Erysipelotrichaceae.* These families are linked to improved bird performance due to their association with the production of short-chain fatty acids. However, the relative abundance of these families tends to decrease during health challenges^22^. Research on salinomycin has yielded inconsistent results, with some studies showing an increase^23^ and others a decrease^24^ in the relative abundance of these bacteria. In this study, the inclusion of SaL did not affect the abundance of these families seven days post-challenge. An increase in the relative abundance of the *Erysipelotrichaceae* family was also observed with the use of OFP_1.5^21^.

*Lactobacillus* spp. act on the immune system by stimulating immune cells to release pro-inflammatory cytokines, such as tumor necrosis factor-alpha (TNF-α), interferon-gamma (IFN-γ), and interleukin-12 (IL-12), triggering an immunomodulatory response during challenge by *Eimeria* spp.. The proliferation of the *Lactobacillus spp.* contributes positively to weight gain, reduces injury scores, and increases mucosal integrity^25^. However, it should be noted that different *Lactobacillus* species have been proven to influence the expression of different cytokines. *L. reuteri*, *L. salivarius*, and *L. johnsonii* are probiotic species that act in different ways on the immune system of animals. *L. reuteri* acts by limiting the gene expression of IFN-Y and TNF-y, inhibiting the production of pro-inflammatory cytokines, including nuclear translocation of nuclear factor-κB (NF-κB)^26^. *L. salivarius* affects intestinal homeostasis, inducing the expression of IFN-γ, IL-12, IL-1β, and TGF-β; therefore, it has a greater capacity to induce a Th1 response. *L. johnsonii* has been studied for its effect on modulating pro-inflammatory cytokines, such as IL-2, IL-8, IL-10, and IFN-γ, and for its action against *C. perfringens*.

Between days 14 and 21, there was a surge in the relative abundance of *C. perfringens*, with the exception of treatments involving OFP_1.5 and Sal_OFP_1.0. This escalation can transpire during a coccidiosis challenge^27,28^. *An Eimeria* infection fosters an ideal environment for the proliferation of *C. perfringens* by liberating cellular proteins and stimulating mucogenesis, which can result in necrotic enteritis and potentially high bird mortality rates^29,30^. In previously published data utilizing the qRT-PCR technique, it was found that the supplementation of functional oils significantly reduced (P < 0.05) the quantity of *C. perfringens* in comparison to the monensin and control groups^31^. Greater resistance in *C. perfringens* strains to virginiamycin than to other antibiotics has been observed ^32^. Alterations in the gut microbiota can lead to changes in metabolic functions. Past studies have associated *Eimeria spp*. infection with an increase in metagenomic genes in cecal content. These genes are involved in pathways related to infectious diseases (viral or parasitic), neurodegenerative diseases, and energy, amino acids, carbohydrates, lipids, cofactors, and vitamin metabolism. They are also involved in the biosynthesis and metabolism of glycans, indicating that *Eimeria* infection can have detrimental effects on nutrient metabolism^33^.

Microbial energy and amino acid metabolic pathways hold significant importance for the host, given that the metabolites of the glycan pathway, namely acetate, propionate, and butyrate, are short-chain fatty acids with crucial health roles^25^. Nitrogen pathways, which include amino acid transport and metabolism, also undergo changes in biogenic amine productio^26^. Notably, there are substantial overlaps between the energy and amino acid metabolic pathways within bacterial metabolism^25^. Salinomycin, as previously discussed, has demonstrated inconsistent effects on bacteria that produce short-chain fatty acids. In this study, these effects were discerned through the metabolic pathways, rather than being directly observed in the bacterial numbers.

Moreover, the application of OFP_1.5 led to an observed increase in the metabolic pathways of terpenes, lipids, and nucleotides compared to anticoccidials. *A negative correlation* has been demonstrated between Lactobacilli and terpenoid metabolism^27,34^. It has been observed that *L. johnsonii* can mitigate the adverse effects of *C. perfringens* and enhance lipid deposition in chicken meat, suggesting a potential role in lipid metabolism ^35^. In this study, the introduction of OFP_1.5 amplified the presence of *L. johnsonii* and diminished that of *C. perfringens*. While further metabolomics-related studies are required, it is postulated that OFP_1.5 impacts the immune system of broilers due to its role in modulating the intestinal microbiota through the alteration of metabolic pathways.

## Conclusion

The physical form of the functional oil blend had no impact on performance and intestinal health parameters. The employment of individual phytogenic additives failed to prevent weight loss in Eimeria-challenged birds, but it mitigated the effects of infection by modulating the intestinal microbiota. A synergistic effect was noted between the functional oil blends and anticoccidials, effectively substituting virginiamycin in the product combination.

### Supplementary Information

Supplementary Figure 1.

Supplementary Figure 2a.

Supplementary Figure 2b.

Supplementary Figure 3a.

Supplementary Figure 3b.

Supplementary Table 1.

Supplementary Table 2.

Supplementary Table 3.

Supplementary Table 4.

## Data Availability

The raw sequences related to this article have been deposited at the Sequence Read Archive (SRA) under the BioProject ID PRJNA882262 (https://dataview.ncbi.nlm.nih.gov/object/PRJNA882262?reviewer=re1c0n188ladm7dbjs0idh8hva).
