# Supplementary material for: Intestinal health of broilers challenged with Eimeria spp. using functional oil blends in two physical forms with or without anticoccidials
Source: Sci Rep. 2023 Sep 5;13:14612. doi: 10.1038/s41598-023-41743-9 (PMC10480430; doi:10.1038/s41598-023-41743-9)
Supplement: Supplementary file 1 — Supplementary Figure 1. [file 41598_2023_41743_MOESM1_ESM.pdf]

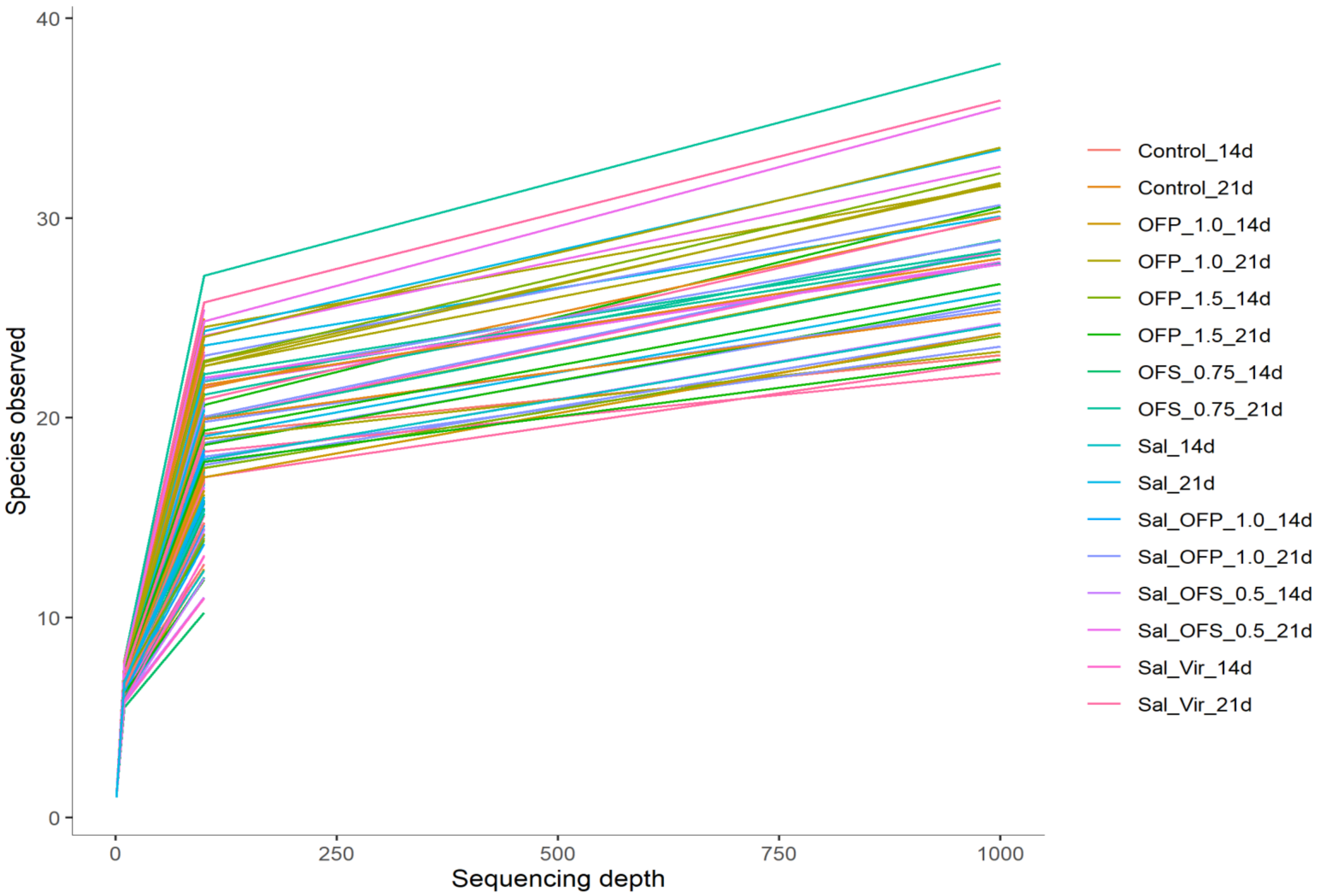

Figure S1- Rarefaction curve of the samples of the experiment composed of eight treatments in two dates, 14 and 21 days of age of broilers.
