# Supplementary material for: Intestinal health of broilers challenged with Eimeria spp. using functional oil blends in two physical forms with or without anticoccidials
Source: Sci Rep. 2023 Sep 5;13:14612. doi: 10.1038/s41598-023-41743-9 (PMC10480430; doi:10.1038/s41598-023-41743-9)
Supplement: Supplementary file 3 — Supplementary Figure 2b. [file 41598_2023_41743_MOESM3_ESM.pdf]

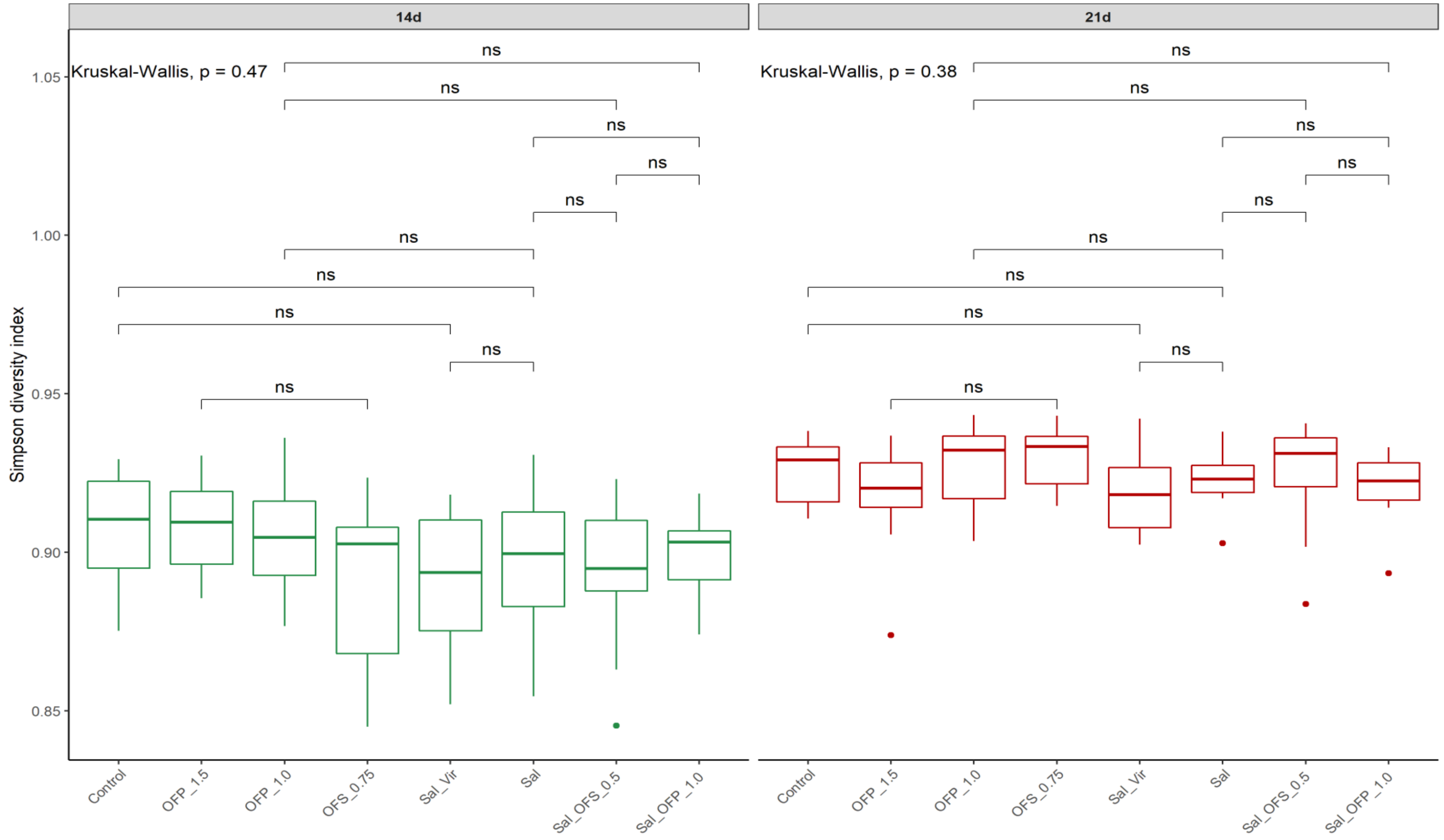

Figure S2b - Comparison of alpha diversity between additives with Simpson n index at 14 and 21 days.

Control: without additive; OFS_0.75: blend of oil functional spray 0.75kg/t; OFP_1.0: blend of oil functional powder 1.0 kg/t; OFP_1.5; blend of oil functional spray 1.5kg/t; Sal: salinomycin 66 ppm; Sal_Vir: virginiamycin 16 ppm and Sal 66 ppm; Sal_OFS_0.5: Sal 66 ppm plus blend of oil functional spray 0.5 kg/t; Sal_OFP_1.0: Sal 66 ppm plus blend of oil functional powder 1.0 kg/t
