# Supplementary material for: Intestinal health of broilers challenged with Eimeria spp. using functional oil blends in two physical forms with or without anticoccidials
Source: Sci Rep. 2023 Sep 5;13:14612. doi: 10.1038/s41598-023-41743-9 (PMC10480430; doi:10.1038/s41598-023-41743-9)
Supplement: Supplementary file 6 — Supplementary Table 1. [file 41598_2023_41743_MOESM6_ESM.docx]

Suplementar table 1. Ingredient formulas and chemical composition of experimental diets according to the rearing period.

| Ingredients, (kg/t) | Starter  (1-21 d) | Grower  (22-35 d) | Finisher  (36-42d) |
| --- | --- | --- | --- |
| Corn | 527.335 | 600.341 | 664.550 |
| Soybean meal 45,5% | 397.204 | 331.794 | 270.000 |
| Soy oil | 34.605 | 32.807 | 33.000 |
| Monodicalcium Phosphate | 10.739 | - | - |
| Dicalcium Phosphate | - | 9.307 | 8.000 |
| Limestone 37% | 14.891 | 12.934 | 12.000 |
| Salt | 4.790 | 4.073 | 3.900 |
| Kaolin ¹ | 2.000 | 2.000 | 2.000 |
| MHA | 4.208 | - | - |
| L – Methionine | - | 2.746 | 2.300 |
| L – Lysine HCL 78% | 1.025 | 1.203 | 1.600 |
| L – Threonine 98% | 0.537 | 0.448 | 0.500 |
| Choline Chloride 60% | 0.616 | 0.597 | 0.700 |
| Vitamin- mineralpremix² | 1.800 | 1.500 | 1.200 |
| BHT | 0.100 | 0.100 | 0.100 |
| Hiphos GT 20000 | 0.100 | 0.100 | 0.100 |
| Rovabio Advanced | 0.050 | 0.050 | 0.050 |
| **TOTAL** | 1,000 | 1,000 | 1,000 |
| Energy and nutrients, % or else as shown | | | |
| ME Poultry, kcal/kg | 3,080 | 3,100 | 3,200 |
| Crude Protein % | 22.83 | 20.00 | 17.70 |
| dLys (%) | 1.22 | 1.15 | 1.00 |
| Av.P (%) | 0.50 | 0.45 | 0.40 |
| Ca (%) | 0.98 | 0.90 | 0.80 |
| Na (%) | 0.22 | 0.19 | 0.18 |

²Vitamin A, UI 14,300; Vitamin D3 UI 5,200; Vitamin E, UI 71.5; Vitamin K3 3.9 mg/kg; Thiamine 3.0 mg/kg; Riboflavin 9.1 mg/kg; Pyridoxyne 5.2 mg/kg; Cyanocobalamin 0.032 mg/kg; Selenium 0.39 mg/kg; Pantothenic acid 15.6 mg/kg; Niacin 78.0 mg/kg;

Folic acid 2.6 mg/kg; Biotin 0.32 mg/kg; Iron 50.0 mg/kg; Zinc 65.0 mg/kg; Manganese 65.0 mg/kg; Copper 10.0 mg/kg; Iodine 1.0 mg/kg;

¹ Each treatment used an antibiotic: salinomycin (66 ppm, MSD Animal Health), virginiamycin (16.5 ppm, Phibro Animal Health, Teaneck, NJ, USA) or different doses of CNSL - Castor oil (0.5 kg/t; 0.75 kg/t; 1.00 kg/t). All antimicrobial doses are recommended at subtherapeutic levels for disease prevention or growth promotion by respective manufacturers.
